# Yeast hydrolysate and exercise ameliorate high-fat diet-induced obesity in C57BL/6 mice

**DOI:** 10.1186/s12906-025-04856-4

**Published:** 2025-04-03

**Authors:** Nari Kim, Yejin Ahn, Eun-Jin Jung, Hyeon-Son Choi, Hyung Joo Suh, Yeok Boo Chang

**Affiliations:** 1 Department of Integrated Biomedical and Life Science, Graduate School, Korea University, Seoul, 02841 Republic of Korea; 2 Research Group of Functional Food Materials, Korea Food Research Institute, Wanju-gun, 55365 Republic of Korea; 3 Department of Food and Biotechnology, Korea University, Sejong, 30019 Republic of Korea; 4 Department of Food Nutrition, Sangmyung University, Hongjimun 2-Gil 20, Jongno-Gu, Seoul, 03016 Republic of Korea; 5 Department of Healthcare Sciences, Graduate School, Transdisciplinary Major in Learning Health Systems, Korea University, Seoul, 02841 Republic of Korea

**Keywords:** Yeast hydrolysate, High-fat diet, Obesity, Lipid metabolism, Antioxidant/anti-inflammatory response

## Abstract

**Background:**

Yeast hydrolysate (YH) has been shown to be effective in inhibiting fat accumulation. This study aimed to investigate the effects of YH and exercise on high-fat diet-induced obesity and their underlying mechanisms in C57BL/6J mice.

**Methods:**

In this study, 6-week-old C57BL/6 mice were divided into 7 groups; a normal group fed a normal chow diet, an HFD group (CON) fed *ad libitum*, an exercise group (15m/min, 30min), an HFD supplemented with low dose of YH (100 mg/kg, YHL), an HFD supplemented with high dose of YH (200 mg/kg, YHH), and a combination of exercise and YHH group. After 12 weeks of HFD and YH administration, white adipocytes and liver tissue were analyzed.

**Results:**

Both YH and exercise significantly reduced the weight of the body and adipose tissues; however, the greatest effect was observed when YH and exercise were combined. Similarly, most reduction in adipocyte size and fat formation in the liver was notable following the combination of YH and exercise. Furthermore, both YH and exercise effectively downregulated lipid synthesis genes, namely fatty acid synthase (FAS), acetyl-CoA carboxylase (ACC), and HMG-CoA reductase (HMGCR), and SREBP1 and SREBP2 proteins. YH and/or exercise also significantly increased the mRNA levels of hormone sensitive lipase (HSL) and carnitine palmitoyltransferase 1 (CPT1), both of which are related to fatty acid oxidation. In addition, pAMPKα and PPARα levels were significantly increased by YH and exercise, with the greatest increase noted following the combination of YH and exercise. Additionally, YH and exercise combined caused the most significant increase in the antioxidant response, as evidenced by nuclear factor erythroid 2-related factor 2 (Nrf2) and superoxide dismutase 1 (SOD1) upregulation at protein and mRNA levels. Their combination also exhibited strongest suppression of inflammatory responses via the downregulation of NF-κB, TNF-α, and IL-1β at protein and mRNA levels. Collectively, YH and exercise combination showed an inhibitory effect on obesity, leading to decreased lipogenesis and increased lipolysis, with favorable regulation of antioxidant and inflammatory responses.

**Conclusion:**

This study suggests the potential use of a combination of physical activity and YH supplementation to control obesity and related metabolic diseases.

**Supplementary Information:**

The online version contains supplementary material available at 10.1186/s12906-025-04856-4.

## Background

Obesity is a complex medical disorder characterized by the excessive accumulation of body fat, which can negatively influence overall health and well-being [1]. It is defined as a body mass index, which is calculated by the weight and height ratio, of 30 kg/m^2^ [1]. This epidemic is caused by a combination of genetic, environmental, and lifestyle factors, including genetic predispositions, dietary habits, reduced physical activity, and certain medications [2]. Obesity is considered a serious health problem because it is implicated in various diseases, such as type 2 diabetes, cancers, and heart diseases, and it can lead to low self-esteem, depression, and social impairment [3]. Fat accumulation in the body results from the dysregulation of fat metabolism, which is an imbalance between fat storage and oxidation [3]. Consumption of processed foods with high sugar and fat content, which is a typical modern diet, leads to an imbalance in metabolism [1]. Fat metabolism is controlled by factors responsible for lipid synthesis and hydrolysis. Sterol regulatory element-binding protein 1 and 2 (SREBP-1/2) are transcription factors and representative regulators of fatty acid and cholesterol synthesis. Sterol regulatory element binding protein 1c (SREBP-1C) is involved in the regulation of fatty acid and triglyceride synthesis, while SREBP-2 is involved in cholesterol metabolism [4]. AMP-activated protein kinase (AMPK) plays crucial roles in cellular energy homeostasis, and its activation in adipose tissues is associated with fat degradation via lipolysis [5]. Peroxisome-proliferator-activated receptor alpha (PPARα) plays an important role in the expression of fat degradation-related genes [6]. Thus, the expression or activation of these regulators plays a decisive role in fat metabolism and homeostasis, influencing obesity development.

Numerous studies have indicated that nuclear factor-κB (NF-κB) is significantly associated with obesity, oxidative stress, and inflammation [7]. Additionally, it has been reported that NF-κB and the Nrf2/HO-1 pathway contribute to obesity and liver steatosis resulting from prolonged high-fat diets[8]. By inhibiting the NF-κB signaling pathway and activating the Nrf2/HO-1 pathway, it is possible to decrease inflammation and alleviate oxidative stress[9].

Yeast is one of the most useful microbes in food science and pharmacology [10]. Microbes, such as *Saccharomyces cerevisiae*, are edible and safe, and therefore used for the fermentation of bread and alcohol, such as beer. Yeast is a good source of protein and its hydrolysates (YH) have been studied for a wide range of research, including their applications [10, 11], nutritional supplements, biodegradable materials (polymers), biofuels, animal feed, health-promoting agents, and cosmetic materials. YH, which contain peptides and amino acids, are produced by the enzymatic hydrolysis of yeast proteins. Yeast-derived peptides have various physiological effects, such as immunomodulatory, antioxidant, anti-inflammatory, antimicrobial, and skin-protective effects [11, 12]. YH reportedly ameliorate obesity [13–15]. This effect was found to be attributed to the active compound 1-methyl-1,2,3,4-tetrahydro-β-carboline-3-carboxylic acid (MTCA). YH contained 0.52 mg/g MTCA and effectively inhibited lipid accumulation via the regulation of fatty acid and cholesterol metabolism in 3T3L1 cells. MTCA-containing YH exhibited an inhibitory effect on fat accumulation by downregulating SREBP-1C and SREBP-2 mRNA in 3T3L1 cells [16]. Moreover, YH have shown to reduce triglyceride content and lipid droplet size in high-sugar diet-fed *Drosophila*. YH affect insulin-like peptide signaling in *Drosophila*, causing changes in neurotransmitter content and diurnal locomotor activity [17]. Thus, the anti-obesity effect of YH appears to be due to MTCA as well as the regulation of genes related to carbohydrate and lipid metabolism [17].

Exercise is a form of physical activity that has numerous benefits for both physical and mental health [18]. Regular and moderate exercise strengthens various organs, such as muscles and the heart, and reduces the risk of chronic diseases, such as type 2 diabetes, blood pressure, certain cancers, and metabolic syndromes [19]. In addition, it improves cognitive function and alleviates depression/anxiety symptoms [19, 20]. Physical activity that involves burning of calories or fat is crucial for weight management [18, 19]. Furthermore, previous research has demonstrated that moderate-intensity treadmill exercise in obesity-induced mice models effectively mitigates the accumulation of cholesterol and oxysterols within mitochondria, thereby exerting significant antioxidant and anti-inflammatory effects.[21]

In this study, the effects of physical activity and/or YH administration on obesity were investigated using high-fat diet (HFD)-fed mice. The synergistic effect of exercise and YH was determined by analyzing the underlying mechanisms, including the regulation of various factors related to fat synthesis, hydrolysis, and anti-oxidation.

## Materials and methods

### Preparation of Yeast Hydorlysate

*Saccharomyces cerevisiae*, supplied by Choheung Chemical, underwent proteolytic enzyme treatment. The resulting hydrolysate, containing peptides with molecular weights under 10 kDa, was isolated using a 10 kDa molecular weight cut-off membrane (Nanofiltration System, SEC, Republic of Korea) and subsequently spray-dried. The final product exhibited a crude protein concentration of 550 mg/g and contained 0.30 mg/g of 1-methyl-1,2,3,4-tetrahydro-β-carboline-3-carboxylic acid (MTCA).

### Cell culture

3T3L1 cells were obtained from the Korean Cell Line Bank (Seoul, Republic of Korea). 3T3L1 cells were cultured in Dulbecco's Modified Eagle's Medium (DMEM) containing 10% fetal calf serum. Differentiation into mature adipocytes was induced for 8 days in DMEM containing 10% fetal bovine serum using differentiation inducers (0.5 mM IBMX, 0.25 μM dexamethasone, and 5 μg/mL insulin) according to a previous method[16]. MTCA, an active compound of YH, was treated at various concentrations every 2 days during the differentiation period. YH containing 0.52 mg/g MTCA was obtained from Neo Cremar., Ltd. (Seoul, Korea).

### Animal maintenance

C57BL/6J mice (6-week-old, male) were purchased from Central Lab. Animal Inc. (Seoul, Korea) and acclimatized to a regular diet for one week. The mice were randomly divided into six groups of ten mice each in a plastic cage. The experimental groups were as follows: a normal group fed a normal chow diet (NOR, Harian Tekiad Global Diets, Madison, WI, USA), an HFD group (CON) fed *ad libitum*, an exercise group, an HFD supplemented with 100 mg/kg YH (YHL), an HFD supplemented with 200 mg/kg YH (YHH), and a combination of exercise and YHH group. To induce obesity, all experimental groups were fed an HFD (Research Diet Inc., New Brunswick, NJ, USA) for 12 weeks (Supplementary Table 1)[22]. The HFD was supplemented with 100 mg/kg YH (YHL) and 200 mg/kg YH (YHH) for 12 weeks, and the mice were administered orally five times a week. Mice in the exercise groups were subjected to treadmill running at a speed of 15 m/min for 30 min, five times a week. The treadmill was set to a 0% incline, and the speed remained consistent throughout each session, with no variations[23]. Mice were housed separately in cages, with three to four mice per cage. The animal housing room was maintained at a temperature of 20 ± 2°C, relative humidity of 60 ± 5%, and a 12-h light-dark cycle. Body weight was measured once weekly. All animal experiments were approved by the Institutional Animal Care and Use Committee of Korea University (KIACUC-2021-0098).

### Blood separation and analysis

The animals were fasted for 12 h and then subjected to isoflurane anesthesia (4 mL/kg) by placing them in a desiccator containing the anesthetic. Then, blood was collected from the femoral artery and transferred to lithium heparin polystyrene tubes. The tubes were centrifuged at 2000 × g for 10 min at 4℃ and the supernatants were collected and stored at −80 ℃ until further analysis. Levels of serum biomarkers, such as glucose, triglycerides, and cholesterol, were analyzed using dry chemistry with the Dri-Chem 3500i analyzer (Fuji Photo Co., Osaka, Japan) and corresponding FUJI DRI-CHEM slides specific for glucose (GLU-P III), triglycerides (TG-P III), and total cholesterol (TCHO-P III).

### Hematoxylin and eosin (H&E) staining

Liver and adipose tissues were fixed in a 10% formaldehyde solution for 24 h, and then dehydrated with increasing concentrations of ethanol starting from 60% ethanol. Then, the tissues were embedded in paraffin, sectioned at 4 μm thickness, stained with H&E, and observed under an optical microscope (Nikon, Tokyo, Japan) at a magnification of 200×. The surface area of epididymal adipose tissue was measured using ImageJ software by determining the distance from edge to edge of adipocytes. In liver tissue, the proportion of white macrovesicular regions was quantified using ImageJ. (National Institutes of Health, Bethesda, MD, USA).

### Real-time PCR analysis

Total RNA was extracted from 3T3L1 cells and tissues (liver, eWAT, iWAT), purified using Trizol reagent (Invitrogen Life Technologies, Grand Island, NY, USA), and quantified using a NanoDrop 1000 spectrophotometer (Thermo Fisher Scientific, Waltham, MA, USA). Subsequently, mRNA expression was analyzed using a CFX96 real-time PCR system (Bio-Rad, Hercules, CA, USA) and SYBR Green PCR kit (Qiagen, Hilden, Germany). Glyceraldehyde-3-phosphate dehydrogenase was used as a reference gene to normalize gene expression. The 2-ΔΔCT method [24] was used to calculate the relative expression. The primer sequences used are listed in Supplementary Table 2.

### Western blotting

Proteins from eWAT tissue (50 mg) were quantified using the bicinchoninic acid method [25], separated using sodium dodecyl sulfate polyacrylamide gel electrophoresis, and transferred onto polyvinylidene fluoride membranes. The membranes were blocked with 5% skim milk and incubated with primary antibodies against SREBP-1C, SREBP-2, PPARα, nuclear factor erythroid 2–related factor 2 (Nrf2), and nuclear factor kappa-light-chain-enhancer of activated B cells (NF-κB) (1:1,000 dilution; Cell Signaling Technology Inc, Danvers, MA, USA) overnight at 4°C. After washing four times for 10 min with Tris-buffered saline with Tween® (TBST), the membranes were incubated with horseradish peroxidase-conjugated anti-rabbit IgG (1:5,000 dilution) for 2 h at room temperature. After four 20 min washes with TBST, the protein bands were detected using a chemiluminescence imaging system (BD Biosciences). Protein levels were quantified using ImageJ software (National Institutes of Health, Bethesda, Maryland).

### Malondialdehyde (MDA) level analysis

MDA levels in homogenized liver tissues were measured using the OxiSelectTM TBARS Assay kit (Cell Biolabs Inc., San Diego, CA, USA) according to the manufacturer’s protocol. Fluorescence was detected at excitation and emission wavelengths of 485 and 535 nm, respectively, using a microplate reader (SpectraMax M3, molecular devices, San Jose, CA, USA).

### Cecal microbiome analysis

Mouse cecum was collected after CO_2_ euthanasia, and genomic DNA was extracted from the feces in the cecum. The cecal microbiome was analyzed using 16S rRNA gene pyrosequencing technology as described previously [26]. Alpha diversity was determined using Chao 1 richness and Shannon diversity indices, and beta diversity was determined using principal coordinate analysis (PCoA). Pearson’s correlation analysis was performed to assess the correlation between lipid metabolism-related indicators and cecal microbiota.

### Statistical analysis

Statistical analyses were performed using SPSS software (version 20.0, Chicago, IL, USA), the normality of the data was assessed using the Shapiro-Wilk test to ensure the validity of the parametric tests, and comparisons among experimental groups were analyzed using one-way analysis of variance and post hoc Tukey’s test. We evaluated the adequacy of the sample size using G*Power software for post-hoc power analysis.

## Results

### Effects of exercise and YH on the weight of the body and white adipose tissues of HFD-induced obese mice

**Fig. 1:**
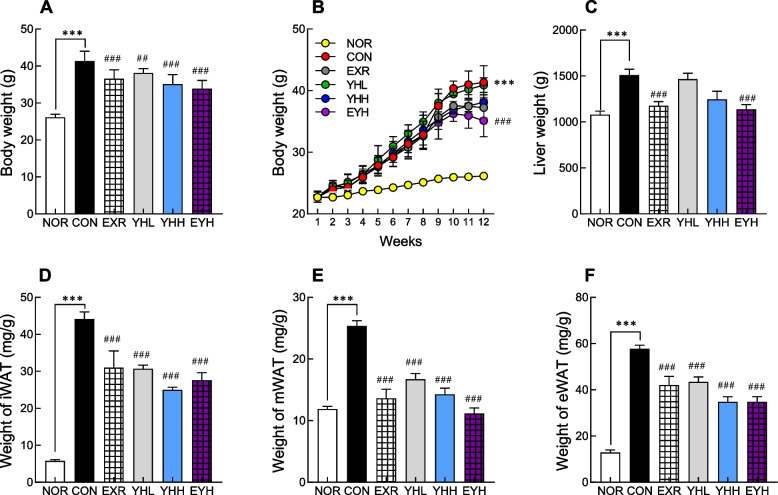
Effect of yeast hydrolysate and exercise on body and tissue weight of HFD-fed C57BL/6 mice. Mice administered with yeast hydrolysate and/or subjected to exercise were examined for body (**A**, **B**) and tissue (**C**-**F**) weight. NOR, normal; CON, control (HFD); EXR, exercise; YH-L/H, low-dose/high-dose yeast protein hydrolysate; EYH, exercise + high-dose yeast protein hydrolysate. Data are presented as the mean ± SD (n=10). Different symbols indicate significant difference; ^***^ *p* < 0.001 vs. NOR group, ^##^ *p* < 0.01, ^###^ *p* < 0.001 vs. CON group based on one-way ANOVA and the Tukey’s test.

The body weight of HFD-fed mice was increased by 60% compared with that of normal group mice (Fig. 1A and B). Physical activity (exercise) using a treadmill and YH significantly reduced the body weight increased by HFD by 11.7% and 16.2%, respectively. Exercise and YHH combined decreased the body weight by 19.2% compared to the CON group (Fig. 1B). These results indicated that physical activity and YH synergistically ameliorated obesity. The weight of liver and inguinal/mesenteric/epididymal white adipose tissues (iWAT/mWAT/eWAT) was significantly decreased by exercise and/or YH supplementation (Fig. 1C-F). Exercise decreased the weight of iWAT, mWAT, and eWAT by 29.8%, 46.3%, and 27.4%, respectively, whereas YHH reduced the weight by 43.7%, 44.7%, and 24.7%, respectively, compared to the CON group. Similarly, exercise and YHH combined decreased the weight of iWAT, mWAT, and eWAT by 37.7%, 55.4%, and 40.0%, respectively (Fig. 1C-E). These results indicated that exercise and YH synergistically exert an inhibitory effect on HFD-induced obesity.

### Effects of exercise and YH on adipose and liver tissue morphology and serum biomarkers

**Fig. 2:**
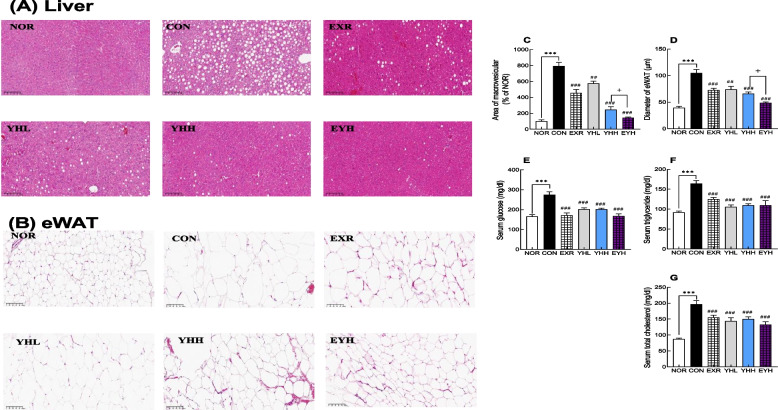
Effect of yeast hydrolysate and exercise on fatty liver, adipose tissue, and serum biomarkers of HFD-fed C57BL/6 mice. Liver and adipose (eWAT) tissues were sectioned and analyzed by H&E staining (**A** and **B**). Macrovesicular lipid area in the liver (**C**) and adipocyte diameter (**D**) were measured. The levels of glucose (**E**), triglycerides (**F**), and total cholesterol (**G**) were measured using a kit. NOR, normal; CON, control (HFD); EXR, exercise; YH-L/H, low-dose/high-dose yeast protein hydrolysate; EYH, exercise + high-dose yeast protein hydrolysate. Data are presented as the mean ± SD (n=10). Different symbols indicate significant difference; ^***^ *p* < 0.001 vs. NOR group, ^##^ *p* < 0.01, ^###^ *p* < 0.001 vs. CON group, ^+^ *p* < 0.05 vs. EYH group based on one-way ANOVA and the Tukey’s test

HFD induced fatty liver development, but exercise and YH effectively suppressed it (Fig. 2A and C). In particular, exercise and YH significantly reduced macrovesicular fatty changes in the liver by 41.6% and 68.1%, respectively, while exercise and YHH combined decreased fatty vacuoles by 81.4% compared to the CON group (Fig. 2C).

Furthermore, HFD increased the size of adipocytes in eWAT. However, exercise and YH significantly decreased adipocyte size in eWAT (Fig. 2B and D). The average diameter of adipocytes was significantly larger (1.6-fold) in the CON group than in the normal group (Fig. 2D). Contrarily, exercise decreased the diameter of adipocytes by 31.2%, whereas YHH reduced it by 37.4% compared with the CON group (Fig. 2D). Furthermore, exercise and YHH combined decreased the adipocyte diameter by 54.3% (Fig. 2D), indicating that their combination reduced adipocyte size more effectively than exercise and YH alone. Collectively, the combination of exercise and YHH effectively mitigated the HFD-induced increase in adipocyte size and fatty liver formation.

Exercise and YHH also decreased serum glucose levels by 35.8% and 25.8%, respectively (Fig. 2E). Furthermore, the combination of exercise and YHH showed a 39.1% reduction in serum glucose levels (Fig. 2E). Similarly, exercise and YH lowered serum triglyceride and cholesterol levels. In particular, exercise reduced triglyceride and cholesterol levels by 23.6% and 21.2%, respectively, whereas YHH showed a 33.8% and 22.6% decrease, respectively (Fig. 2F and G). Exercise and YHH combined lowered triglyceride and cholesterol levels by 34.1% and 31.3% (Fig. 2F and G). These results indicated that the combination of exercise and YHH improved serum biochemical values related to lipid accumulation.

### Effects of exercise and YH on the expression of fat synthesis genes

**Fig. 3:**
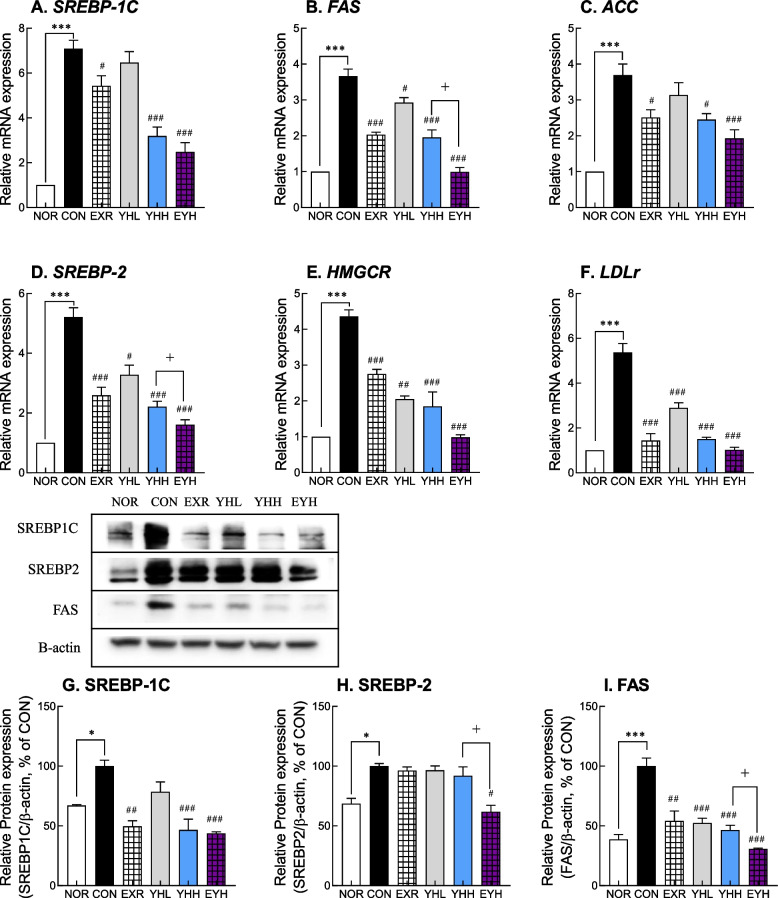
Effects of yeast hydrolysate and exercise on lipid synthetic factors in the liver and adipose tissues of HFD-fed C57BL/6 mice. Expression of lipid synthesis genes was determined using real-time PCR (**A**-**F**) in liver tissues. Protein expression of SREBP-1C/2 and FAS in adipose tissues was examined using western blotting (**G**-**L**). NOR, normal; CON, control (HFD); EXR, exercise; YH-L/H, low-dose/high-dose yeast protein hydrolysate; EYH, exercise + high-dose yeast protein hydrolysate. Data are presented as the mean ± SD (n=10). Different symbols indicate significant difference; ^*^ *p* < 0.05, ^**^ *p* < 0.01, ^***^ *p* < 0.001 vs. NOR group, ^#^ *p* < 0.05, ^##^ *p* < 0.01, ^###^ *p* < 0.001 vs. CON group, ^+^ *p* < 0.05 vs. EYH group based on one-way ANOVA and the Tukey’s test

In liver, mRNA levels of SREBP-1C, a master gene involved in fatty acid synthesis, were significantly reduced by exercise and YH (Fig. 3A). Exercise, YHH, and their combination downregulated SREBP-1C by 23.2%, 54.9%, and 64.9%, respectively (Fig. 3A). SREBP-1C protein levels were also reduced following exercise and YHH administration. Additionally, the combination of exercise and YHH downregulated SREBP-1C by 56.32% (Fig. 3G). The gene expression of fatty acid synthase (FAS) and acetyl-CoA carboxylase (ACC), both of which are controlled by SREBP-1C, was decreased by 43.9% and 31.9%, respectively, in exercised animals (Fig. 3B and C). YH administration also reduced FAS and ACC expression in a dose-dependent manner; YHL and YHH decreased the gene expression of FAS by 20.2% and 46.5%, respectively, and ACC by 14.3% and 34.3%, respectively (Fig. 3B and C). Consistent with these results, FAS protein abundance was significantly decreased after YH administration and exercise, and the combination of YHH and exercise reduced FAS protein levels by 69.18% compared to the CON group (Fig. 3I). The expression of SREBP-2, a transcription factor involved in cholesterol biosynthesis, was significantly decreased by exercise and YH. In particular, exercise, YHL, and YHH decreased SREBP-2 gene expression by 50.4%, 38.2%, and 57.7%, respectively, compared to the CON group (Fig. 3D). SREBP-2 gene expression was the most strongly inhibited (68.8%) by the combination of exercise and YHH compared to exercise and YHH alone (Fig. 3D). However, exercise and YH did not exhibit a significant reduction in SREBP-2 protein level, but both of them combined significantly reduced SREBP-2 protein level by 38.19% (Fig. 3H). Furthermore, mRNA expression of HMG-CoA reductase (HMGCR) and low-density lipoprotein receptor (LDLR) was significantly suppressed by exercise and YH administration; in particular, exercise showed a 57.3% and 72.5% reduction in HMGCR and LDLR mRNA expression, respectively, while YHH showed a 53.4% and 72.2% reduction, respectively (Fig. 3E and F). Exercise and YHH combined decreased HMGCR and LDLR mRNA levels by 72.2% and 81.3%, respectively (Fig. 3E and F). These results indicated that exercise and YH administration significantly suppressed fatty acid and cholesterol synthesis, but their combination showed the greatest effect in suppressing the expression of fatty acid and cholesterol biosynthesis genes.

**Fig. 4:**
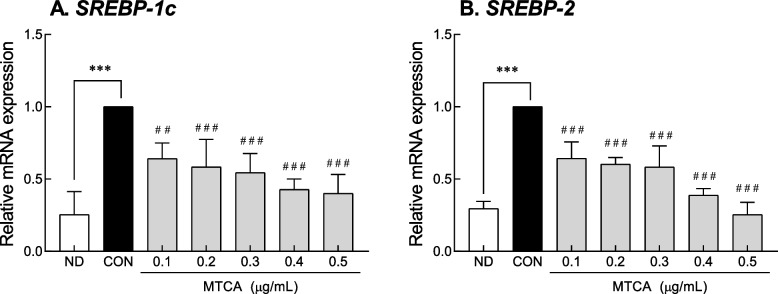
Effect of MTCA on lipid synthesis factors in 3T3L1 cells. ND: non-differentiated 3T3L1 cells, CON: differentiated 3T3L1 cells, MTCA: 1-Methyl-1,2,3,4-tetrahydro-β-carboline-3-carboxylic Acid, SREBP: sterol regulatory element-binding protein. Data are presented as the mean ± SD. *** p < 0.001 vs. ND group, and ## *p* < 0.01, ### *p *< 0.001 vs. CON group based on one-way ANOVA and the Tukey’s test

The effect of MTCA, an active compound of YH, on fatty acid synthesis was evaluated in 3T3L1 preadipocytes (Fig. 4). The CON group significantly increased the mRNA expression of SREBP-1C (3.94-fold) and SREBP-2 (3.41-fold) compared to the undifferentiated ND group. MTCA treatment significantly inhibited the expression of SREBP-1C and SREBP-2 in a dose-dependent manner compared to the CON group. These results suggest that the lipid accumulation inhibitory activity of YH is attributed to MTCA.

### Effects of exercise and YH administration on the expression of NADPH synthesis genes

**Fig. 5:**
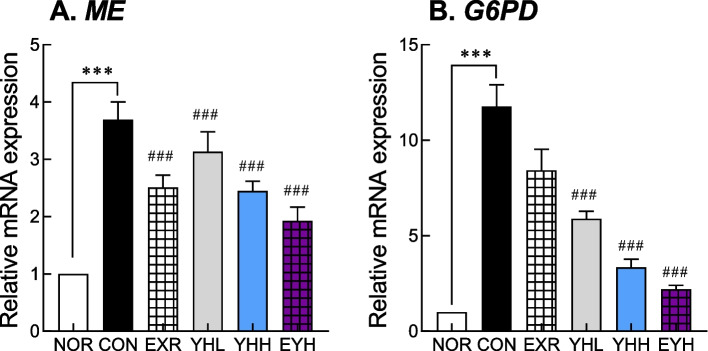
Effect of yeast hydrolysate and exercise on malic enzyme and G6PD mRNA expression in HFD-fed C57BL/6 mice. mRNA expression of ME (**A**) and G6PD (**B**) in liver was analyzed using real-time PCR. NOR, normal; CON, control (HFD); EXR, exercise; YH-L/H, low-dose/high-dose yeast protein hydrolysate; EYH, exercise + high-dose yeast protein hydrolysate. Data are presented as the mean ± SD (n=10). Different symbols indicate significant difference; ^***^ *p* < 0.001 vs. NOR group, ^###^ *p* < 0.001 vs. CON group based on one-way ANOVA and the Tukey’s test

In liver tissue, malic enzyme (ME) and glucose-6 phosphate dehydrogenase (G6PD), which are involved in NADPH synthesis, were downregulated by exercise and YH. Exercise suppressed ME and G6PD mRNA expression by 64.8% and 28.6%, respectively (Fig. 5) and YHH by 59.7% and 71.3%, respectively. However, exercise and YHH combination decreased ME and G6PD gene expression by 68.7% and 81.9%, respectively (Fig. 5). This result indicated that exercise and YH effectively suppressed the synthesis of NADPH, which is used as a co-enzyme during fatty acid and cholesterol biosynthesis.

### Effects of exercise and YH administration on the expression of fat hydrolysis genes

**Fig. 6:**
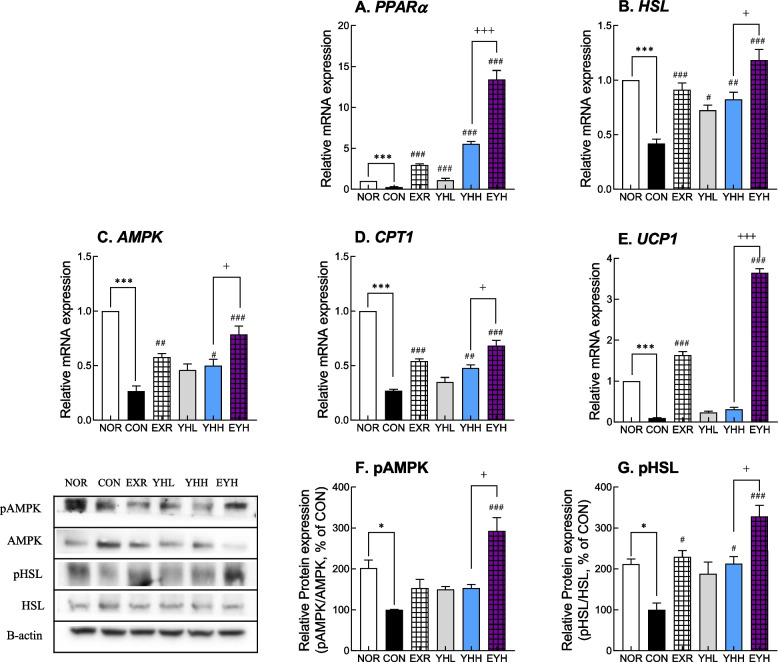
Effect of yeast hydrolysate and exercise on lipolytic factors in HFD-fed C57BL/6 mice. mRNA expression of lipolysis factors were analyzed using real-time PCR in liver (**A**-**C**) and iWAT (**D**-**E**). Protein expression of pAMPK (**F**) and pHSL (**G**) was analyzed using western blotting in eWAT. NOR, normal; CON, control (HFD); EXR, exercise; YH-L/H, low-dose/high-dose yeast protein hydrolysate; EYH, exercise + high-dose yeast protein hydrolysate. Data are presented as the mean ± SD (n=10). Different symbols indicate significant difference; ^*^ *p* < 0.05, ^**^ *p* < 0.01, ^***^ *p* < 0.001 vs. NOR group, ^#^ *p* < 0.05, ^##^ *p* < 0.01, ^###^ *p* < 0.001 vs. CON group, ^+^ *p* < 0.05, ^+++^ *p* < 0.001 vs. EYH group based on one-way ANOVA and the Tukey’s test

At the mRNA level, HFD downregulated PPARα and UCP1 in iWAT, as well as hormone-sensitive lipase (HSL) and carnitine palmitoyltransferase 1 (CPT1) in the liver, all of which are related to lipid hydrolysis or oxidation. The mRNA level of PPARα, which is a nuclear receptor and promotes the expression of genes involved in lipid catabolism, increased by 9-fold after exercise and 3.2- and 16.7-fold after YHL and YHH administration, respectively (Fig. 6A). Moreover, YHH and exercise combined upregulated PPARα mRNA by 40-fold compared to the CON group (Fig. 6A). Furthermore, exercise, YHL, and YHH significantly increased HSL mRNA levels by 2.17-fold, 69.5%, and 93.4%, respectively, compared to the CON group (Fig. 6B). The combination of exercise and YHH caused the highest increase in HSL mRNA levels (2.78-fold). In addition, YH and exercise activated HSL and AMPK by increasing their phosphorylation (Fig. 6E and F). However, HSL and AMPK were the most activated by the combination of YH and exercise than by exercise and YH alone. YHH and exercise combination reversed the HFD-mediated reduction in the phosphorylation/total protein ratio by 3.28- and 2.92-fold, respectively (Fig. 6E and F). CPT1, which is responsible for fatty acid oxidation, showed an expressional pattern similar to that of PPARα and HSL (Fig. 6D). Exercise and YHH elevated CPT1 mRNA expression by 2.0- and 1.3-fold, respectively, compared to the CON group (Fig. 6D). In addition, UCP1 was upregulated 13.3- and 2.3-fold after exercise and YHH, respectively (Fig. 6E). The highest upregulation of CPT1 and UCP1 observed was when exercise was combined with YHH administration, where the expression of both genes was increased by 2.5- and 29-fold, respectively (Fig. 6D and E). This result indicated that exercise and YH combined was most effective in upregulating lipid hydrolysis genes.

### Effects of exercise and YH administration on anti-oxidative response

**Fig. 7:**
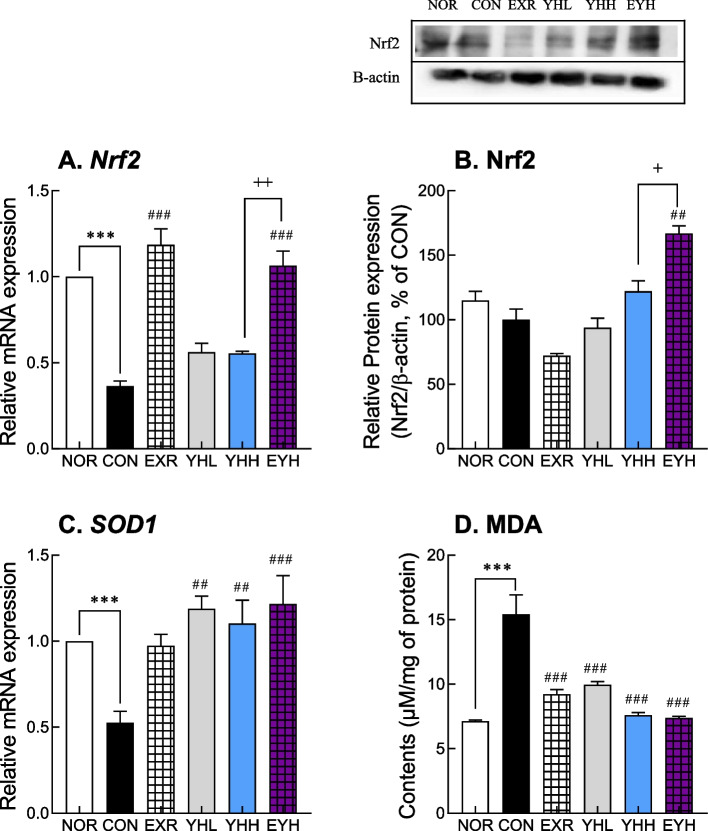
Effect of yeast hydrolysate and exercise on antioxidant responses in HFD-fed C57BL/6 mice. Nrf2 mRNA (**A**) and protein (**B**) levels were analyzed in liver using real-time PCR and western blotting, respectively. mRNA expression of SOD1 and MDA was measured using real-time PCR and TBARS assay, respectively (**C** and **D**). NOR, normal; CON, control (HFD); EXR, exercise; YH-L/H, low-dose/high-dose yeast protein hydrolysate; EYH, exercise + high-dose yeast protein hydrolysate. Data are presented as the mean ± SD (n=10). Different symbols indicate significant difference; ^*^ *p* < 0.05, ^**^ *p* < 0.01, ^***^ *p* < 0.001 vs. NOR group, ^#^ *p* < 0.05, ^##^ *p* < 0.01, ^###^ *p* < 0.001 vs. CON group, ^+^ *p* < 0.05, ^++^ *p* < 0.01 vs. EYH group based on one-way ANOVA and the Tukey’s test

Nrf2, a master gene for the antioxidant response, and superoxide dismutase 1 (SOD1), an antioxidant gene, were downregulated by the HFD in liver tissue. However, exercise and YH reversed this effect (Fig. 7A and C). In particular, exercise upregulated Nrf2 mRNA by 3.2-fold, while YH showed a 50% increase in Nrf2 gene expression compared to the CON group (Fig. 7A). At the protein level, exercise and YHH combined upregulated Nrf2 by 1.67-fold (Fig 7B). Similarly, SOD1 gene expression was significantly increased by exercise and YH by 85.2% and 2.2-fold, respectively, compared to that of the CON group (Fig. 7C). The combination of exercise and YHH increased SOD1 mRNA expression by 2.32-fold compared with the CON group (Fig. 7C). Furthermore, HFD increased MDA levels, but exercise and YH significantly reversed this effect. In particular, exercise, YHH, and their combination decreased MDA levels by 40.6%, 50.7%, and 51.1% compared to the CON group (Fig. 7D). These results showed that exercise and YHH administration activated antioxidant responses to ameliorate obesity.

### Effects of exercise and YH administration on the inflammatory response

**Fig. 8:**
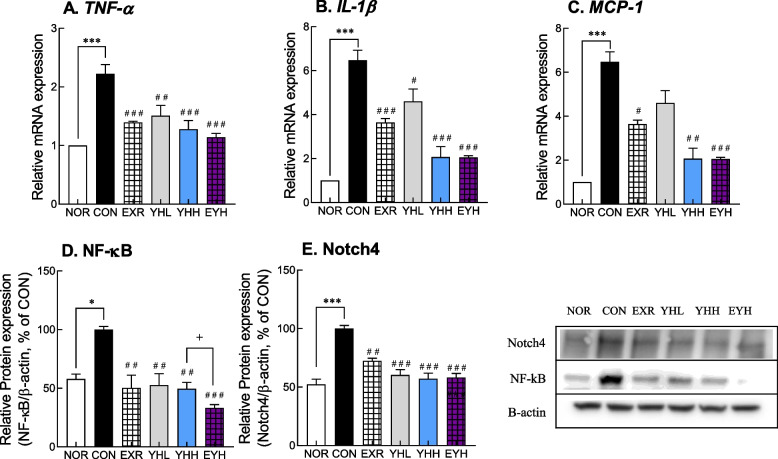
Effect of yeast hydrolysate and exercise on the inflammatory response in HFD-fed C57BL/6 mice. mRNA levels of TNF-α (**A**), IL-1β (**B**), and MCP-1 (**C**) were analyzed using real-time PCR in eWAT. NF-κB (**D**) and Notch4 (**E**) protein level was determined using western blot analysis in eWAT. NOR, normal; CON, control (HFD); EXR, exercise; YH-L/H, low-dose/high-dose yeast protein hydrolysate; EYH, exercise + high-dose yeast protein hydrolysate. Data are presented as the mean ± SD (n=10). Different symbols indicate significant difference; ^*^ *p* < 0.05, ^**^ *p* < 0.01, ^***^ *p* < 0.001 vs. NOR group, ^#^ *p* < 0.05, ^##^ *p* < 0.01, ^###^ *p* < 0.001 vs. CON group, ^+^ *p* < 0.05 vs. EYH group based on one-way ANOVA and the Tukey’s test

HFD increased the mRNA expression of inflammatory cytokines, namely tumor necrosis factor-alpha (TNF-α) and interleukin-1 beta (IL-1β) in eWAT tissue. The mRNA expression of TNF-α increased by HFD was decreased by exercise and YH administration; exercise downregulated TNF-α by 37.8% and YHH by 37.3% compared to the CON group (Fig. 8A). The combination of exercise and YHH showed a 48.6% reduction in TNF-α mRNA expression (Fig. 8A). The pattern of IL-1β gene expression was similar to that of TNF-α gene expression. Furthermore, exercise and YHH administration downregulated IL-1β mRNA by 43.7% and 67.3%, respectively, and their combination downregulated it by 75% (Fig. 8B). In addition, NF-κB, a master regulator of an inflammatory response, was downregulated by both exercise and YHH administration by 49.7% and 51.0%, respectively. The most significant downregulation of NF-κB was by the combination of exercise and YHH (67.0%; Fig. 8D). Similarly, the protein expression of Notch4 was significantly reduced by 42.8% and 41.9% with YHH and EYH administration, respectively (*p*<0.001, Fig. 8E). These results indicated that exercise and YH combined was the most effective in inhibiting inflammatory cytokines.

### Effects of exercise and YH administration on the composition of cecal microbiota

**Fig. 9:**
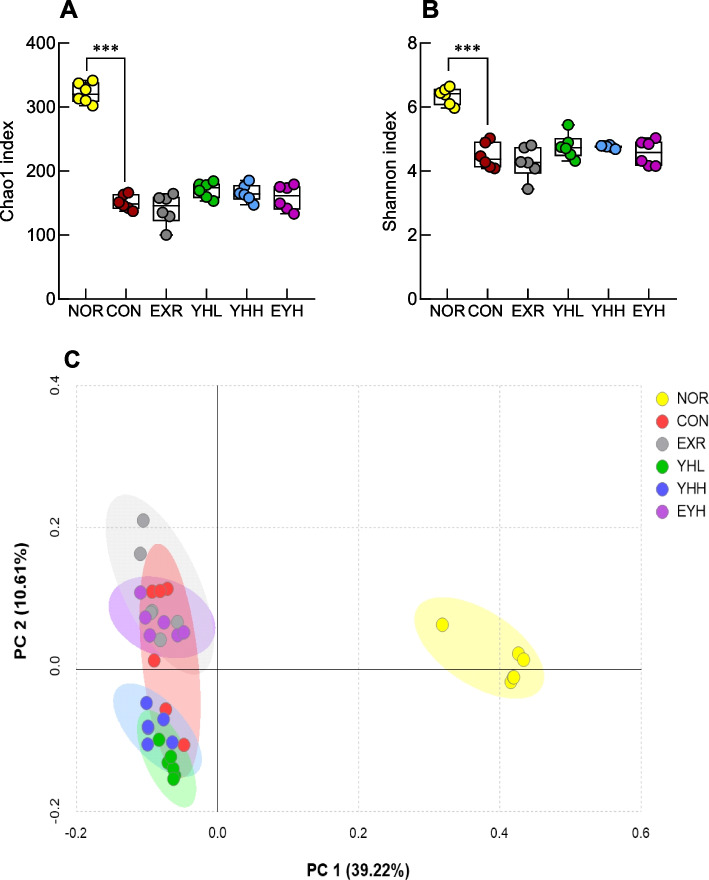
Effect of yeast hydrolysate and exercise on gut microbiome diversity. Chao1 richness (**A**) and Shannon diversity (**B**) were analyzed using 16S rRNA gene pyrosequencing. Beta diversity was expressed using principal coordinate analysis (PCoA) (**C**). NOR, normal; CON, control (HFD); EXR, exercise; YH-L/H, low-dose/high-dose yeast protein hydrolysate; EYH, exercise + high-dose yeast protein hydrolysate. Data are presented as the mean ± SD (n=10). Different symbols indicate significant difference; ^***^ *p* < 0.001 vs. NOR group based on one-way ANOVA and the Tukey’s test

The diversity and richness of the gut microbiome were assessed using the Chao 1 (Fig. 9A) and Shannon (Fig. 9B) indices and found that the HFD significantly reduced these indices (*p*<0.001). The beta diversity of the gut microbiota was assessed using PCoA, which revealed that the HFD-fed groups showed marked differences in microbial distribution compared to the NOR group (Fig. 9C). The intestinal microbial diversity of the EXR and EYH groups was similar to that of the CON group.

**Fig. 10:**
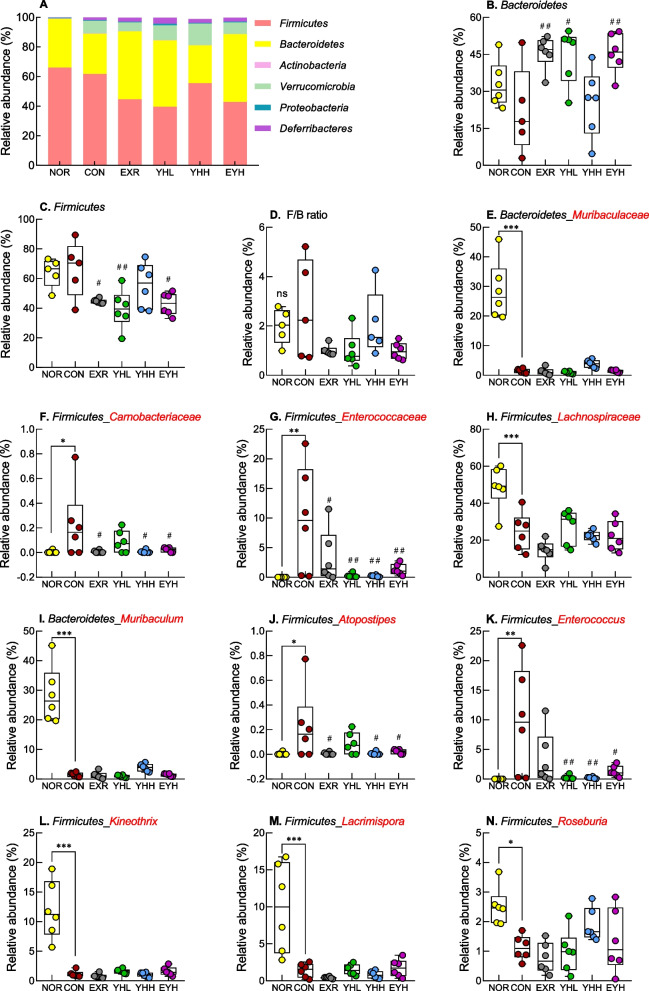
Effects of yeast hydrolysate and exercise on the gut microbiome composition at the phylum level in HFD-fed C57BL/6 mice. The gut microbiome was analyzed using rRNA gene sequencing at the phylum (**A**-**D**), family (**E**-**H**), and genus (**I**-**N**) levels. NOR, normal; CON, control (HFD); EXR, exercise; YH-L/H, low-dose/high-dose yeast protein hydrolysate; EYH, exercise + high-dose yeast protein hydrolysate. Data are presented as the mean ± standard error of the mean. ^*^ *p* < 0.05, ^**^ *p* < 0.01, ^***^ *p* < 0.001 vs. NOR group, ^#^ *p* < 0.05, ^##^ *p* < 0.01, ^###^ *p* < 0.001 vs. CON group based on one-way ANOVA and the Tukey’s test. ns, non-significant (*p* > 0.05)

The relative abundance of individual microorganisms at the phylum and genus levels was analyzed. At the phylum level (Fig. 10A), the abundance of *Firmicutes* (39.58–66.05%) and *Bacteroidetes* (25.43–45.81%) was the highest, followed by that of *Verrucomicrobia* (0.00–14.52%), *Deferribacteres* (0.26–4.21%), *Proteobacteria* (0.38–1.05%), and *Actinobacteria* (0.10–0.31%) in all experimental groups. The combination of YH and exercise significantly reversed the abundance of the phyla *Bacteroidetes* (*p*<0.01; Fig. 10B) and *Firmicutes* (*p*<0.05; Fig. 10C) altered by the HFD. The *Firmicutes*/*Bacteroidetes* (F/B) ratio was increased in the CON group and decreased in the YH and exercise combination groups; however, the changes were not significantly different (Fig. 10D).

**Fig. 11:**
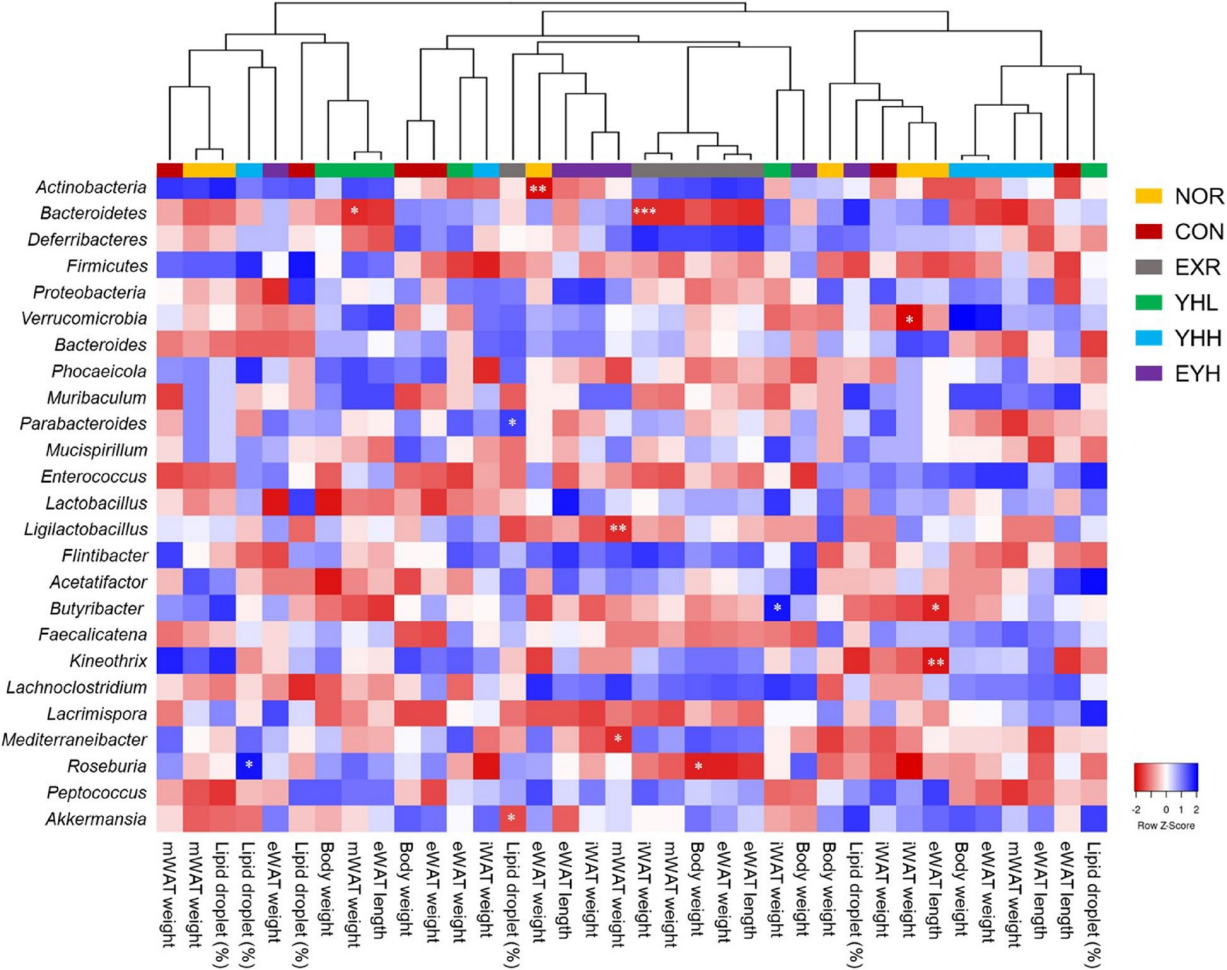
Pearson correlation analysis between intestinal microbiota and obesity-related biomarkers. NOR, normal; CON, control (HFD); EXR, exercise; YH-L/H, low-dose/high-dose yeast protein hydrolysate; EYH, exercise + high-dose yeast protein hydrolysate. Significant differences between the parameters are indicated as ^*^ *p* < 0.05 and ^**^ *p* < 0.01

At the family level, the abundance of *Muribaculaceae*, which belongs to the *Bacteroidetes* phylum, was significantly lower in the CON group than in the NOR group (Fig. 10E). In addition, the relative abundance of *Carnobacteriaceae*, *Enterococcaceae*, and *Lachnospiraceae* families, belonging to the *Firmicutes* phylum, was significantly different between the NOR and CON groups (Fig. 10F-H). Moreover, the combination of YH and exercise significantly reduced the relative abundance of *Carnobacteriaceae* (*p*<0.05) and *Enterococcaceae* (*p*<0.01) increased by the HFD. At the genus level, the HFD decreased the abundance of *Muribaculum* (family, *Muribaculaceae*; Fig. 10I) and increased that of *Atopostipes* (family, *Carnobacteriaceae*; Fig. 10J) and *Enterococcus* (family, *Enterococcaceae*; Fig. 10K). Additionally, HFD significantly lowered the relative abundance of *Kineothrix*, *Lacrimispora*, and *Roseburia*, which belong to the *Lachnospiraceae* family, compared to the NOR group (Fig. 10L-N). In contrast, the combination of YH and exercise significantly suppressed the HFD-mediated increase in *Atopostipes* and *Enterococcus* abundance (*p*<0.05). The correlation between obesity-related biomarkers and the cecal microflora was confirmed using Pearson’s correlation analysis (Fig. 11). The *Bacteroidetes* phylum was negatively correlated with the weight of iWAT and mWAT. The phylum *Verrucomicrobia* was negatively correlated with iWAT weight and serum triglycerides, whereas the genus *Akkermansia* was negatively correlated with lipid droplets (%) (Fig. 11 and Supplementary Fig. 3). The genera *Butyribacter* and *Kineothrix* were negatively correlated with eWAT diameter. Additionally, the *Roseburia* genus showed a negative correlation with the body weight and serum triglyceride and glucose levels (Supplementary Fig. 1).

## Discussion

The incidence of obesity among individuals of different ages, sexes, and socioeconomic backgrounds has been steadily increasing worldwide for decades. The management of obesity involves diet and physical activity regulation, medical interventions, and bariatric surgery. Obesity and excessive fat accumulation can be regulated by changing the lifestyle, such as diet and physical activity, and addressing causes of weight gain [2, 27]. One of the most prevalent ways to control metabolic diseases, including obesity, is exercise, which is a form of physical activity [27]. Regular physical activity may decrease the risk of various chronic diseases, including heart disease, type 2 diabetes, some cancers, obesity, mental health disorders (such as depression), and neurological conditions, while enhancing well-being and quality of life [28]. Several studies have investigated the effects of physical activity (exercise) on obesity [29–32]. Although the effect of physical activity on weight management or body mass index are controversial [33], the main effect of physical activity on weight is via the activation of the AMPK pathway in muscles and other tissues [34]. Consistently, our study results showed that exercise and exercise and YH combined activated AMPK. AMPK activation during exercise increases the activity of HSL, a triglyceride lipolytic enzyme, via its phosphorylation [35, 36], which is consistent with our study findings. During exercise, lipolysis enzymes, such as HSL, are involved in the release of fatty acids from adipocytes and transfer to skeletal muscles via blood vessels for mitochondrial β-oxidation, a fatty acid degradation process [37]. The entry of fatty acids into mitochondria is mediated by CPT1, which governs β-oxidation [38]. In this study, exercise increased CPT1 gene expression, which is in line with that of a previous study reporting an increase in CPT1 activity with exercise [39]. CPT gene expression is regulated by the nuclear factor PPARα, which promotes the expression of fatty acid oxidation-related genes [40]. PPARα forms a heterodimer with the nuclear factor retinoid X receptor, which then binds to peroxisome proliferator response elements, a DNA region, in target genes, such as CPT1. Exercise reportedly improves metabolism by activating PPARα [41]. Swimming mitigates renal fibrosis via PPARα activation [42]. Consistently, our study showed an exercise-mediated increase in PPARα mRNA levels. Exercise-mediated weight loss has primarily been attributed to lipid hydrolysis; however, lipid synthesis is also regulated by exercise. Recently, an 8-week endurance training program reduced the activity of ACC, an enzyme involved in fatty acid synthesis, in visceral adipose tissues [43]. Consistent with this finding, our data showed that exercise effectively suppressed the expression of fatty acid synthesis genes, including ACC. On the contrary, Holland et al. (2016) showed that 6-week exercise increased ACC protein expression in the visceral WAT in rats [44]. May et al. (2017) reported that exercise differentially regulates ACC expression in white and brown adipose tissues, in that, ACC is upregulated in WAT but downregulated in brown adipose tissues [45]. The effect of exercise on lipid synthesis appears to depend on the intensity and duration of exercise as well as on the type of adipose tissue. Fatty acid synthesis is regulated by the transcription factor SREBP-1C, whose nuclear activation promotes the expression of fatty acid synthesis genes, such as FAS and ACC. However, the effect of exercise on SREBP-1C gene expression is controversial. Acute exercise increases SREBP-1C gene expression in adipose tissues [46], whereas resistance training decreases it; however, the differences were not significant Although the effect of exercise on SREBP-1C is obscure, AMPK activation is known to suppress SREBP-1C mRNA and protein expression. Our results showed that exercise significantly downregulated SREBP-1C. The synergy between YH and exercise is likely due to their combined effects on AMPK activation and SREBP inhibition. While exercise primarily activates AMPK through increased energy expenditure, YH components may enhance this activation by providing additional bioactive molecules that stimulate AMPK. The dual inhibition of SREBPs by both interventions leads to a more pronounced decrease in lipogenic gene expression, resulting in reduced lipid synthesis and storage.

Exercise is also known to decrease cholesterol levels [47]. Our results are in line with these findings. SREBP-2 promotes the expression of cholesterol synthesis genes. A recent study showed that the infusion of irisin, a myokine released during exercise, downregulated SREBP-2 [48]. Similarly, the present study revealed that exercise effectively downregulated HMG-CoA reductase, the main enzyme involved in cholesterol synthesis, at the mRNA level. Collectively, exercise positively regulated lipid metabolism in a favorable way.

Inflammation is an obesity-associated response. In obesity, adipose tissues expand and not only store fat, but also release bioactive substances, including adipokines and cytokines, to regulate metabolism [49]. In addition, leptin and adiponectin levels are often imbalanced in obesity; leptin level is elevated, and adiponectin level is reduced, thereby contributing to chronic inflammation and insulin resistance [50]. Furthermore, adipose tissues recruit immune cells, such as macrophages, which produce pro-inflammatory cytokines, including IL-6 and TNF-α. This accumulation of macrophages in adipose tissues induces an inflammatory response [49]. In the current study, obesity-induced inflammatory response was effectively ameliorated by exercise and YH administration by decreasing IL-1β and TNF-α levels. Therefore, the exercise/YH-mediated reduction in the inflammatory response is thought to be attributed to the reduction in adipose tissues. NF-κB, a master gene for inflammatory response, promotes the expression of pro-inflammatory cytokines, such as IL-1β and TNF-α. Aerobic exercise suppresses NF-κB signaling to alleviate post-stroke depression [51]. Here, exercise and YH suppressed the obesity-induced inflammatory response by downregulating NF-κB protein.

Notch4 is a key signaling protein involved in vascular and lymphatic formation and inflammatory response regulation [52]. In the obese state, inflammatory cytokines such as TNF-α and IL-6 increase in adipose tissue, a hypoxic environment is established, and vascular and lymphatic circulation functions tend to decline [53]. These changes may lead to the hyperactivation of the Notch signaling pathway, potentially causing endothelial dysfunction, impaired lymphangiogenesis, and metabolic dysregulation in adipose tissue [54]. This study confirmed that Notch4 protein expression was significantly increased in a HFD-induced obese mouse model and that YH and treadmill exercise effectively suppressed the expression. These findings indicate that Notch4 plays a significant role in obesity and metabolic disorders and that exercise and specific nutritional interventions can serve as effective strategies for its regulation.

Obesity also induces an oxidative stress response, which is defined as an imbalance between reactive oxygen species (ROS) production and antioxidant defense systems [55]. Excessive ROS production can damage cellular components, including proteins, lipids, and DNA, thereby contributing to the development of insulin resistance, endothelial dysfunction, and cellular damage [56]. Enlarged adipocytes release higher levels of cytokines or adipokines, leading to increased ROS production [55]. However, increased ROS level is neutralized by an antioxidant defense system that involves ROS-scavenging enzymes, such as SOD, catalase, and Gpx [57]. Nrf2 is a crucial regulator of the antioxidant response and protects against cellular oxidative stress [58]. Under normal conditions, Nrf2 is sequestered in the cytoplasm by forming a complex with Kelch-like ECH-associated protein 1 (Keap1), facilitating its degradation through the proteasomal degradation process to maintain low Nrf2 levels in the cytoplasm [58]. However, under oxidative stress or exposure to electrophilic substances, Nrf2 is released from the complex following modification by Keap1, leading to the inhibition of Nrf2 degradation. Nrf2 then translocates to the nucleus, where it binds to the antioxidant response element in the promoter region of antioxidant or detoxifying genes to maintain redox balance. Thus, Nrf2 activation leads to the upregulation of various antioxidant/phase II detoxifying genes [59]. In this study, exercise activated Nrf2. Exercise-mediated activation of Nrf2 is caused by physical activity-induced generation of ROS, which can act as a signaling molecule in various cellular processes. Regular and adaptive exercise lead to Nrf2 activation, which is associated with health benefits [59]. However, excessive and prolonged ROS production without sufficient adaptation can lead to oxidative stress and cellular damage [56]. In addition to moderate exercise, certain compounds can also promote Nrf2 activation. Several studies have reported the synergistic effect of exercise/physical activity and Nrf2-activating compounds [60, 61]. Similarly, this study showed that the combination of exercise and YH had a greater effect on the elevation of Nrf2 levels compared to exercise and YH alone.

Studies on the relationship between YH and obesity are limited compared to those on the effects of exercise on obesity. Jung et al. (2012, 2014) demonstrated that YH significantly reduced the body weight of obese adults and HFD-induced obese mice [14, 15]. In line with these findings, our study found that YH inhibited ME and G6PD, which are responsible for the synthesis of NADPH, a cofactor for lipid synthesis. However, the comprehensive and fundamental mechanisms underlying the effects of YH have rarely been studied. The current study showed that YH regulates lipid metabolism, including synthesis and lipolysis, and that YH-mediated anti-obesity effects involve various signaling molecules. Among two diastereoisomers (1S,3S and 1R,3S) contained in yeast hydrolysate, MTCA is an active substance that is also contained in commercial fruits, especially citrus fruits [62]. In a previous study, it was reported that the treatment of MTCA isolated from garlic to 3T3-L1 cells significantly regulated genes related to adipogenesis and lipid metabolism, resulting in anti-adipogenic effects [63]. In addition, we reported that when MTCA was treated as a single compound, it significantly decreased the mRNA levels of SREBP-1C and SREBP-2 in 3T3-L1 cells, as well as regulated NADPH-synthesizing genes, affecting fat accumulation during adipocyte differentiation [16]. In high-sugar-induced obese flies, exposure to yeast hydrolysates affected lipid accumulation by modulating the mRNA expression of *Drosophila* insulin-like peptides related to food preference and metabolism as well as lipid metabolism-related gene and the content of the inhibitory neurotransmitter gamma-aminobutyric acid [17].

Obesity is associated with gut microbiome dysbiosis, and obese individuals have an elevated F/B ratio compared to healthy individuals owing to the decreased abundance and diversity of the gut microbiome and changes in the major phyla *Firmicutes* and *Bacteroidetes* [64]. Similarly, in this study, the HFD altered the distribution of the microbiome, but the combination of YH and exercise reversed the imbalance between *Firmicutes* and *Bacteroidetes*, lowering the F/B ratio. Short-chain fatty acids (SCFAs) produced by intestinal microorganisms are involved in lipid and carbohydrate metabolism and the regulation of energy homeostasis [64]. Here, HFD decreased the abundance of SCFA-producing bacteria (*Kineothrix*, *Lacrimispora*, and *Roseburia*). Moreover, a deficiency in butyrate-producing bacteria, such as *Roseburia* and *Eubacterium*, has been observed in obese individuals [65]. Butyrate suppresses inflammation by inhibiting the NF-κB pathway and contributes to alleviating insulin resistance and obesity through endocrine regulation [66]. Furthermore, there is growing evidence that exercise is associated with changes in gut microbiota. Exercise restores gut dysbiosis by increasing the abundance of *Bacteroidetes* and ameliorating HFD-induced reduction in butyrate production [67]. Interestingly, we found that the combination of exercise and YH markedly inhibited HFD-induced *Alistipes* enrichment. The genus *Alistipes* is an anaerobic bacterium observed at a higher abundance in obese than in lean individuals [68]. Liu et al. [69] reported that 12 weeks of exercise significantly altered the abundance of *Alistipes putredinis* in prediabetic men. In particular, a decrease in *A. putredinis* abundance has been reported in individuals whose insulin sensitivity was improved by exercise.

In this study, both YH and exercise significantly ameliorated obesity. However, the exercise performed in this study exhibited a superior effect on obesity compared to the low dose of YH (YHL) and a similar effect to that of the high dose of YH (YHH), contingent upon the experimental biomarkers. The current study only analyzed the effect of moderate level of exercise, which corresponded to VO_2_ max 60%. As the impact of exercise on obesity and related metabolism depends on the intensity of exercise, other intensities or durations of exercise should be investigated for obesity and its metabolic mechanisms in future studies. To strengthen the conclusions of our study, which confirmed the YH-mediated anti-obesity effects through the regulation of lipid metabolism and associated signaling molecules, future investigations should aim to identify the active anti-obesity compounds in YH. Additionally, applying the same experimental approach in alternative obesity models, such as high-sugar diet-induced obesity, would be valuable. This would help validate that the observed effects are not restricted to high-fat diets.

Our research primarily focused on the impacts of YH and treadmill exercise on lipid synthesis and breakdown, along with their antioxidant and anti-inflammatory effects. Future studies should aim to elucidate the precise mechanisms by which Notch4 signaling is modulated and explore the effects of various exercise and dietary interventions on its expression and function. Furthermore, exploring membrane-bound signaling pathways is crucial, as they play significant roles in lipid metabolism and energy homeostasis. This broader investigation could uncover novel mechanisms and therapeutic strategies for addressing obesity and related metabolic disorders.

## Conclusion

The current study demonstrated the individual and combined effects of YH and exercise on obesity. YH and exercise contributed to the suppression of obesity at similar levels. YH and exercise combined showed a synergistic effect on the amelioration of obesity, with a more favorable regulation of lipid metabolic signaling. The inflammatory and oxidative responses were also ameliorated by the combination of YH and exercise. Furthermore, YH and exercise combined reversed HFD-induced alterations in gut microbiome, such as an increase in the F/B ratio and the abundance of *Atopostipes* and *Enterococcus*. This study indicates that a combination of YH and physical activity may be effective in ameliorating obesity and related metabolic diseases.

## Supplementary Information

Supplementary Material 1.

## Data Availability

The data that support the findings of this study are available from the corresponding author upon reasonable request.

## Abbreviations

ACC: acetyl-CoA carboxylase
AMPKα: AMP-activated protein kinase alpha
CPT1: Carnitine palmitoyltransferase 1
G6PD: Glucose-6 phosphate dehydrogenase
GAPDH: Glyceraldehyde-3-phosphate dehydrogenase
HSL: Hormone-sensitive lipase
HMGCR: HMG-CoA reductase
FAS: Fatty acid synthase
IL-1β: Interleukin-1 beta
LDLR: Low-density lipoprotein receptor
MCP-1: Monocyte chemoattractant protein-1
MTCA: 1-methyl-1,2,3,4-tetrahydro-β-carboline-3-carboxylic acid
ME: Malic enzyme
Nrf2: nuclear factor erythroid 2–related factor 2
SOD1: superoxide dismutase 1
SREBP: sterol regulatory element-binding proteins
TNF-α: Tumor necrosis factor-alpha
UCP1: uncoupling protein 1
YH: yeast hydrolysate
