# Supplementary material for: Yeast hydrolysate and exercise ameliorate high-fat diet-induced obesity in C57BL/6 mice
Source: BMC Complement Med Ther. 2025 Apr 3;25:126. doi: 10.1186/s12906-025-04856-4 (PMC11967133; doi:10.1186/s12906-025-04856-4)

NFkB

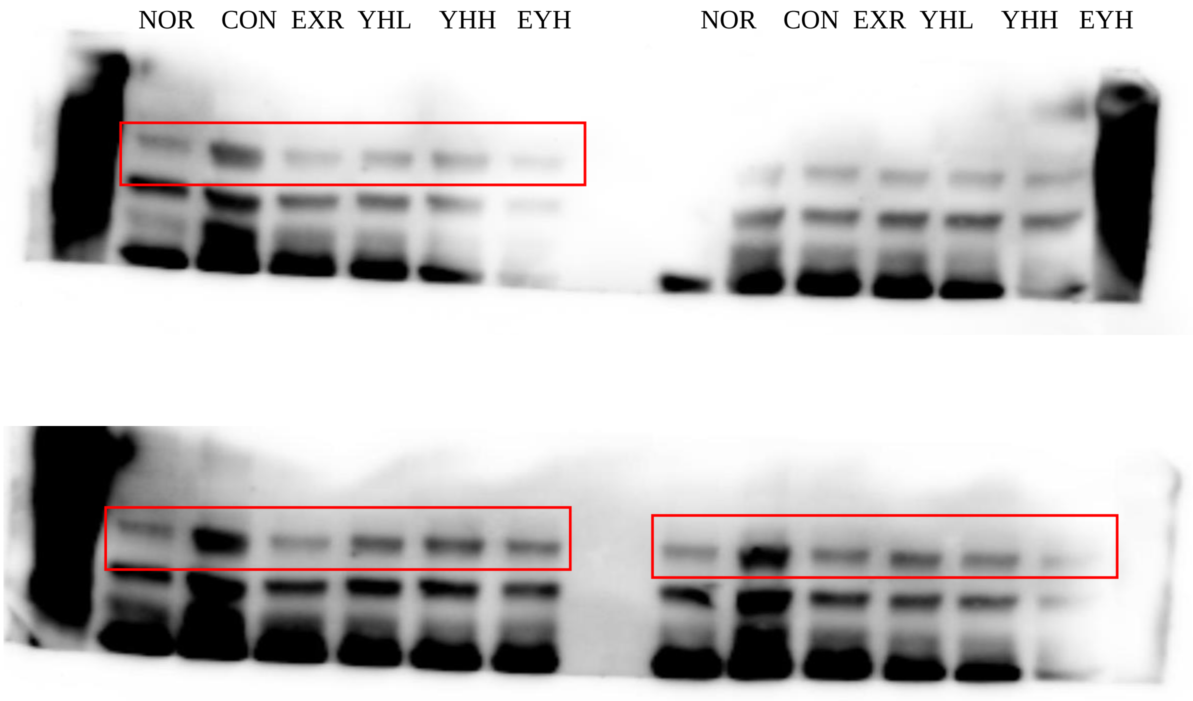

SREBP2

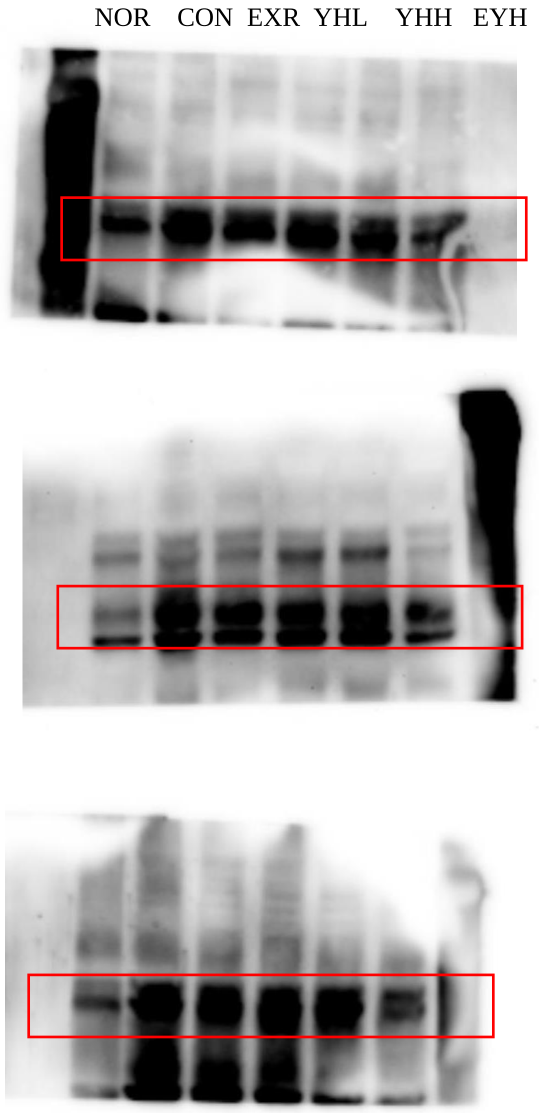

SREBP1C

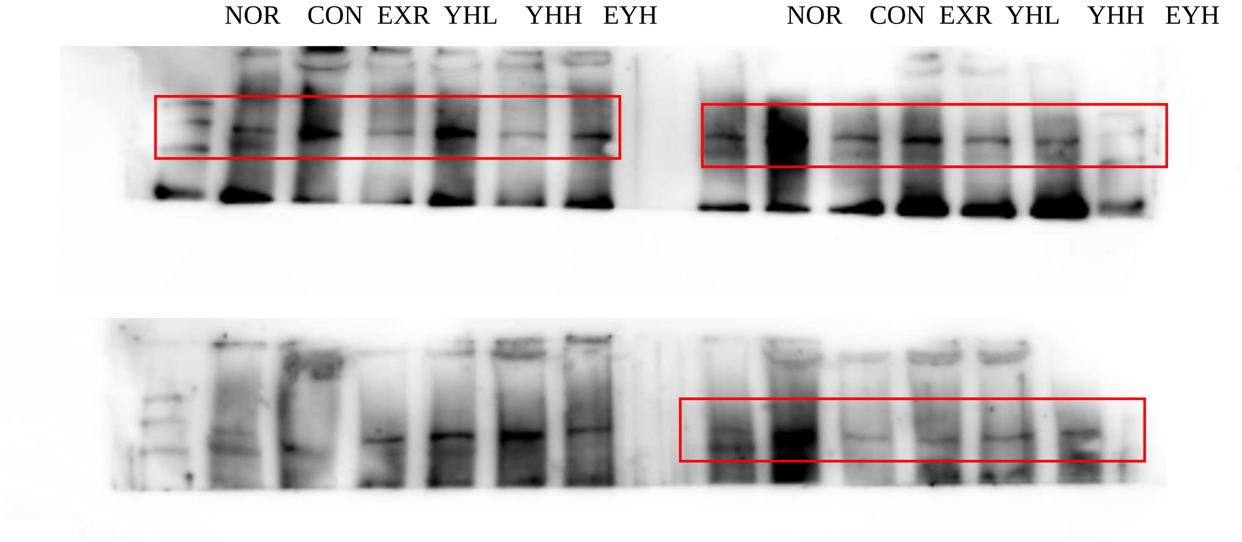

Nrf2

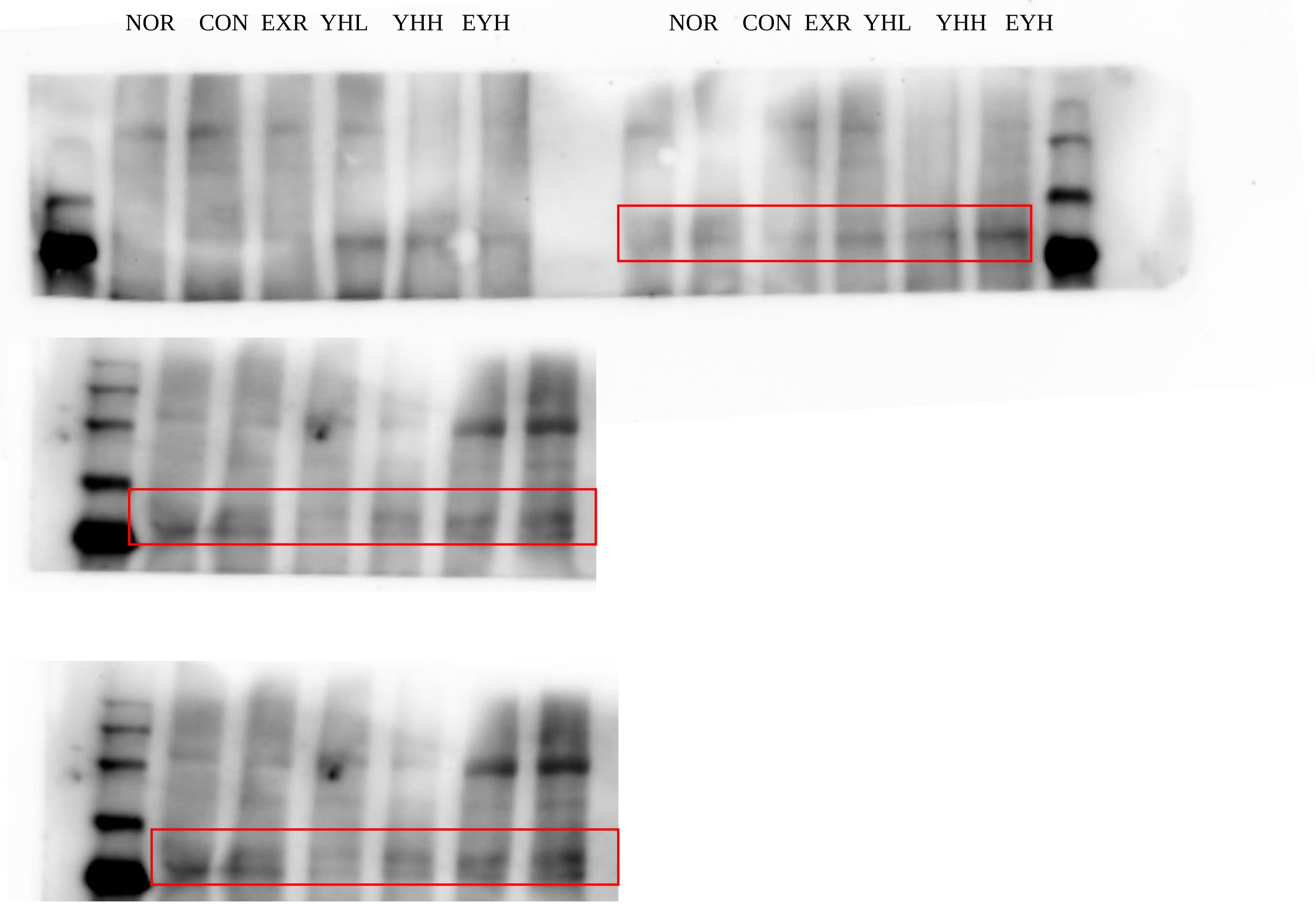

FAS

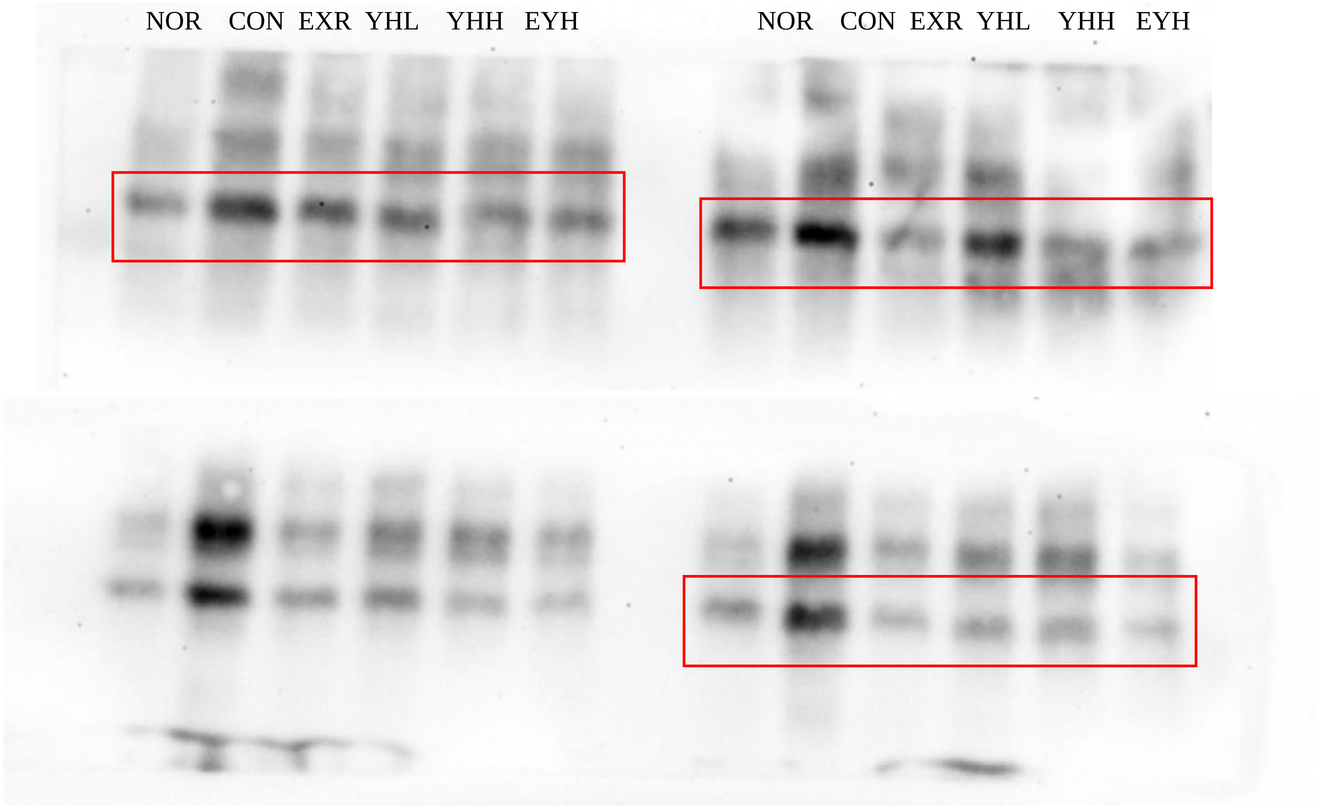

pHSL

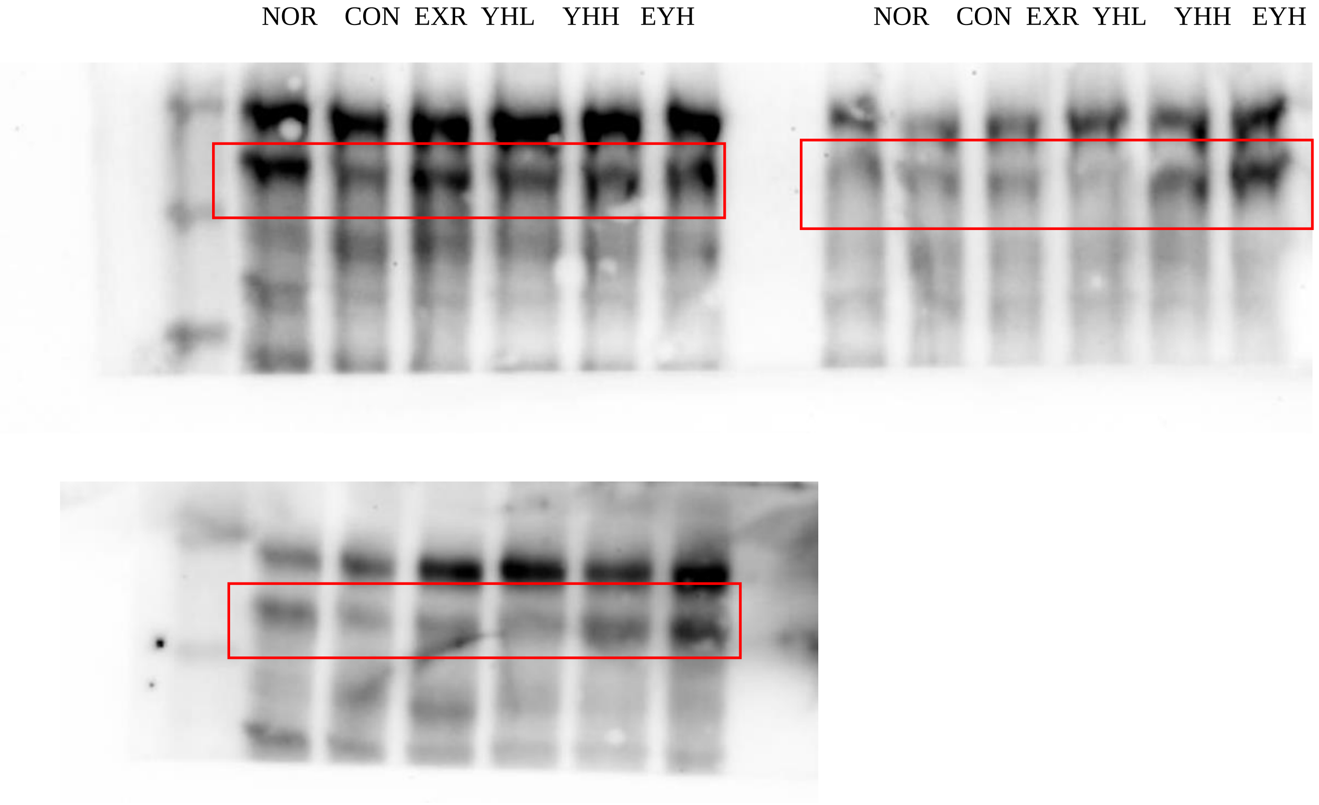
NOR CON EXR YHL YHH EYH
NOR CON EXR YHL YHH EYH

HSL

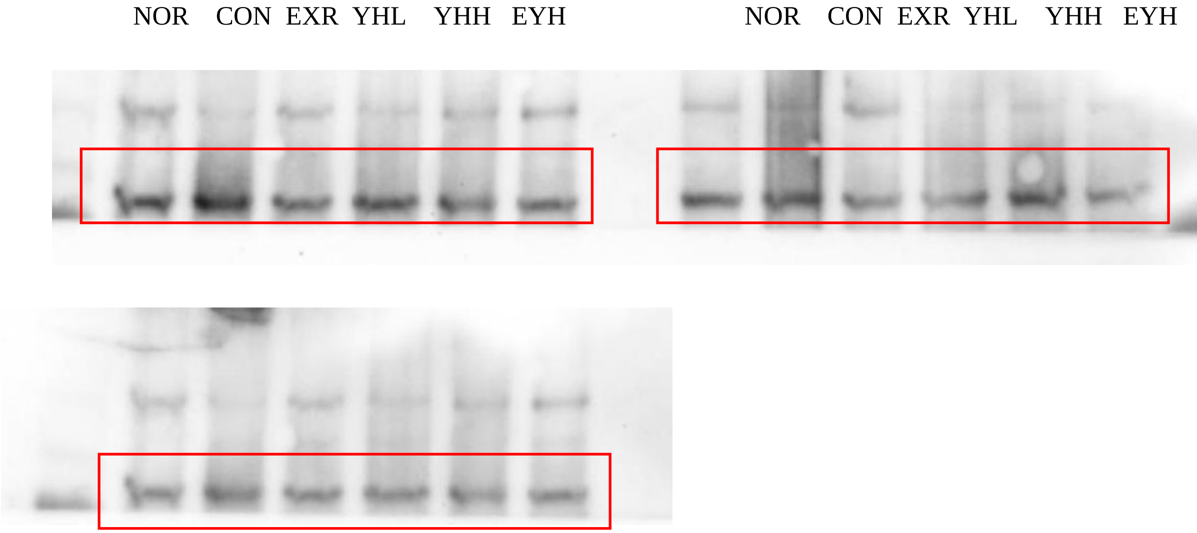
NOR CON EXR YHL YHH EYH
NOR CON EXR YHL YHH EYH

pAMPK

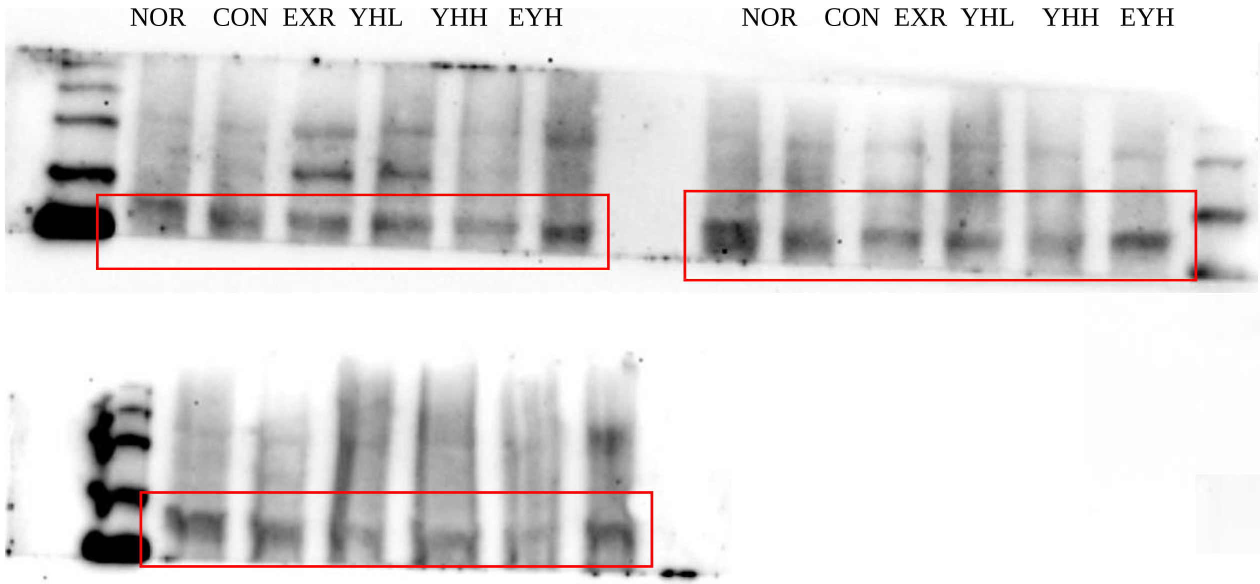
NOR CON EXR YHL YHH EYH
NOR CON EXR YHL YHH EYH

AMPK

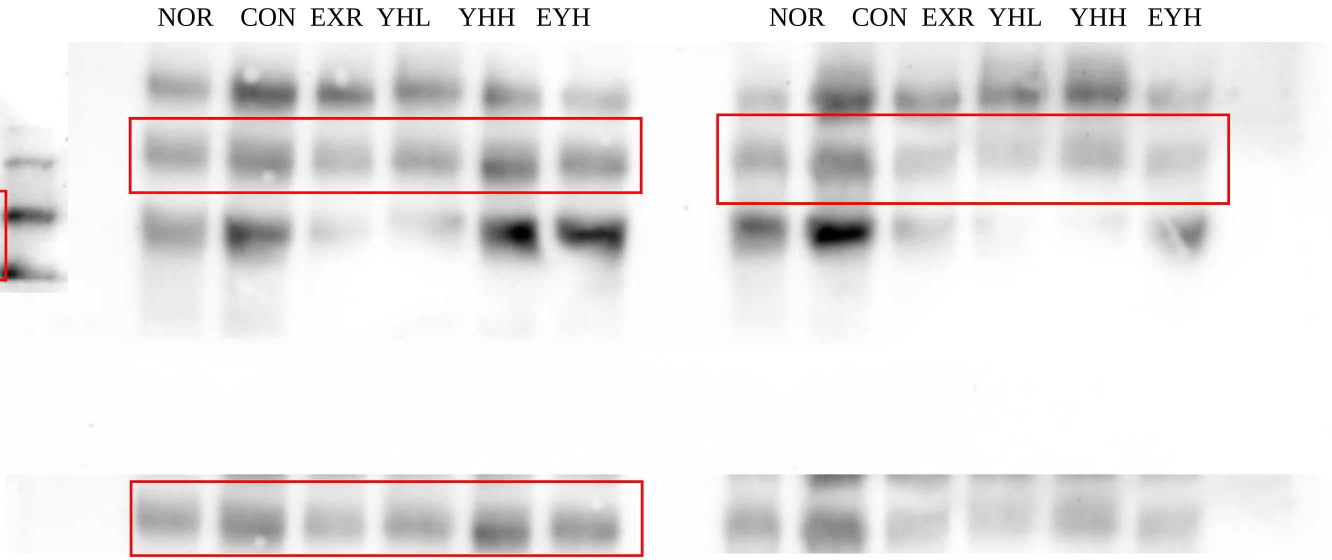
NOR CON EXR YHL YHH EYH
NOR CON EXR YHL YHH EYH

β-actin

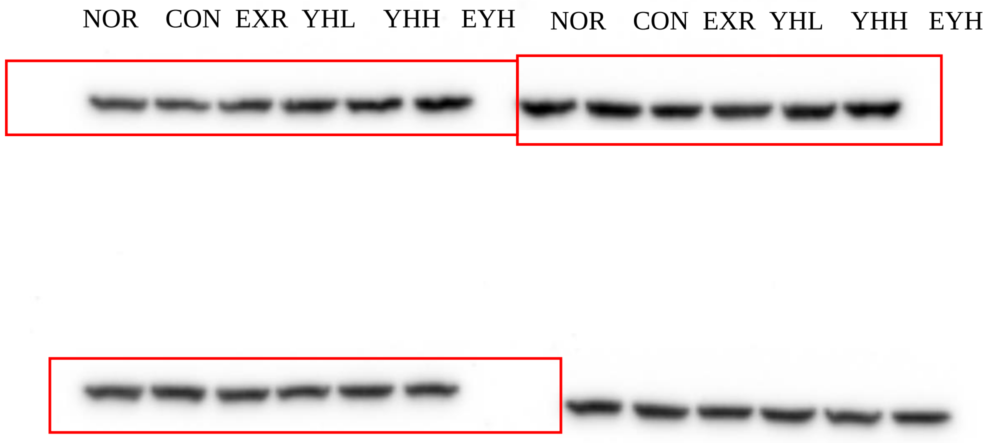

Supplement: Supplementary file 1 — Supplementary Material 1. [file 12906_2025_4856_MOESM1_ESM.pdf]
